# Targeted p120-Catenin Ablation Disrupts Dental Enamel Development

**DOI:** 10.1371/journal.pone.0012703

**Published:** 2010-09-16

**Authors:** John D. Bartlett, Justine M. Dobeck, Coralee E. Tye, Mirna Perez-Moreno, Nicole Stokes, Albert B. Reynolds, Elaine Fuchs, Ziedonis Skobe

**Affiliations:** 1 Department of Cytokine Biology, Forsyth Institute, Boston, Massachusetts, United States of America; 2 Department of Developmental Biology, Harvard School of Dental Medicine, Boston, Massachusetts, United States of America; 3 Centro Nacional de Investigaciones Oncologicas, Madrid, Spain; 4 Howard Hughes Medical Institute, Rockefeller University, New York, New York, United States of America; 5 Department of Cancer Biology, School of Medicine, Vanderbilt University, Nashville, Tennessee, United States of America; University of North Carolina at Charlotte, United States of America

## Abstract

Dental enamel development occurs in stages. The ameloblast cell layer is adjacent to, and is responsible for, enamel formation. When rodent pre-ameloblasts become tall columnar secretory-stage ameloblasts, they secrete enamel matrix proteins, and the ameloblasts start moving in rows that slide by one another. This movement is necessary to form the characteristic decussating enamel prism pattern. Thus, a dynamic system of intercellular interactions is required for proper enamel development. Cadherins are components of the adherens junction (AJ), and they span the cell membrane to mediate attachment to adjacent cells. p120 stabilizes cadherins by preventing their internalization and degradation. So, we asked if p120-mediated cadherin stability is important for dental enamel formation. Targeted p120 ablation in the mouse enamel organ had a striking effect. Secretory stage ameloblasts detached from surrounding tissues, lost polarity, flattened, and ameloblast E- and N-cadherin expression became undetectable by immunostaining. The enamel itself was poorly mineralized and appeared to be composed of a thin layer of merged spheres that abraded from the tooth. Significantly, p120 mosaic mouse teeth were capable of forming normal enamel demonstrating that the enamel defects were not a secondary effect of p120 ablation. Surprisingly, blood-filled sinusoids developed in random locations around the developing teeth. This has not been observed in other p120-ablated tissues and may be due to altered p120-mediated cell signaling. These data reveal a critical role for p120 in tooth and dental enamel development and are consistent with p120 directing the attachment and detachment of the secretory stage ameloblasts as they move in rows.

## Introduction

Dental enamel development progresses through defined stages that can be observed by the changing morphology of the enamel organ that covers the developing murine tooth. The ameloblasts of the enamel organ are a single layer of tall, columnar cells that are responsible for enamel development at their apical cell surface and attach to the stratum intermedium of the enamel organ at their basal end. During the secretory stage of enamel development, enamel matrix proteins are secreted and long thin crystallites form normal to the secretory surface of the ameloblasts. These crystallites will eventually span the distance between the dentin and the enamel surface. The crystallites are bundled into rods, each rod is the mineralized trail left by one ameloblast [1], [2]. Rows of ameloblasts will move past each other to form the decussating enamel rod pattern that is characteristic of rodent dental enamel [3], [4], [5].

Once the enamel layer reaches its full thickness, the ameloblasts transition (transition stage) into shortened maturation stage cells. It is during the maturation stage that ameloblasts actively remove the previously secreted proteins from the enamel matrix as the enamel crystallites grow in width and thickness [reviewed in [6], [7]]. As ameloblasts proceed through the stages of enamel development, the enamel matures from a soft cheese-like substance into its final hardened form. Mature enamel is the hardest substance in the body. Its hardness is intermediate between that of iron and carbon steel, but has higher elasticity [8]. Once the enamel is fully mature, ameloblasts regress and become part of the reduced enamel organ that covers and protects the completed enamel surface until the tooth erupts [9]. Therefore, ameloblasts progress through defined developmental stages that require intercellular contact and communication. A benefit of studying tooth development in rodents is that their incisors continuously erupt. Thus, rodent incisors continuously progress through all developmental stages of tooth development and are excellent models to study the necessary cellular interactions as development progresses.

Adherens junctions perform multiple functions including: initiation and stabilization of cell-cell adhesion, regulation of the actin cytoskeleton, intracellular signaling and transcriptional regulation. The adherens junction is composed of several classes of protein including cadherins and catenins. Cadherins are transmembrane proteins with extracellular domains that provide important adhesive contacts between neighboring cells. The extracellular domains connect through homotypic trans-pairing between cadherins on adjacent cells and the intracellular domains of cadherins are linked to the actin cytoskeleton by the catenins. The functional activity of the adherens junction is mediated in part by cadherin and catenin family members including E- and N-cadherins and p120-catenin [reviewed in [10]].

p120-catenin binds to an intracellular cadherin domain near the cell membrane termed the “juxtamembrane domain” (JMD). Mutation of the E-cadherin JMD demonstrated that p120-E-cadherin interaction was required for high level cell adhesiveness [11], [12] and it was also shown that binding of p120 to the JMD prevents cadherins from becoming internalized and degraded [13], [14], [15], [16]. Previously, we demonstrated that E-cadherin, β-catenin and p120-catenin are expressed in ameloblasts during dental enamel development [17]. Additionally, adherens junctions were previously identified along the possible sliding interface of adjacent secretory stage ameloblast rows [18]. Therefore, adherens junctions may be important for enamel formation and we sought to determine if p120 ablation adversely affected dental enamel development.

Here we directly examine the *in vivo* consequences of p120 ablation on tooth development. Conventional p120 knockout in mice is embryonic lethal [19], [20], but conditional knockout (cKO) mice containing floxed p120 and the Cre recombinase linked to the keratin-14 promoter (K14-Cre p120-cKO) survive [21]. The K14 promoter directs Cre expression to ectodermally derived tissues, including the enamel organ [22], [23]. Dental enamel in K14-Cre p120-cKO mice was severely malformed. The enamel on the erupted teeth was rough, grainy and not well mineralized. The enamel eroded off the occlusal surfaces with time, presumably from the stress of normal mastication. Interestingly, the teeth appeared to develop normally until the secretory stage of enamel development. Thus, the epithelial-mesenchymal interactions necessary for early tooth development were largely unaffected by p120 ablation. However, once the ameloblasts reached the secretory stage, they detached from the dentin and/or stratum intermedium. The cells then became disorganized, flattened, and lost their polarity. This loss of ameloblast organization is consistent with the resulting rough, grainy and hypo-mineralized dental enamel. p120 appeared to stabilize both E- and N-cadherin on ameloblasts so cadherin loss likely plays a role in the observed ameloblast detachment. Importantly, mice mosaic for p120 ablation did have teeth with areas of well-formed healthy enamel, demonstrating that the loss of p120 directly affects tooth development and that the observed enamel defects were not a secondary effect of p120 ablation. These observations indicate an essential role for p120 in stabilizing ameloblast intercellular interactions during the secretory stage of dental enamel development.

## Results

### Cadherins and p120 Are Expressed in the Enamel Organ During Dental Enamel Development

**Figure 1:**
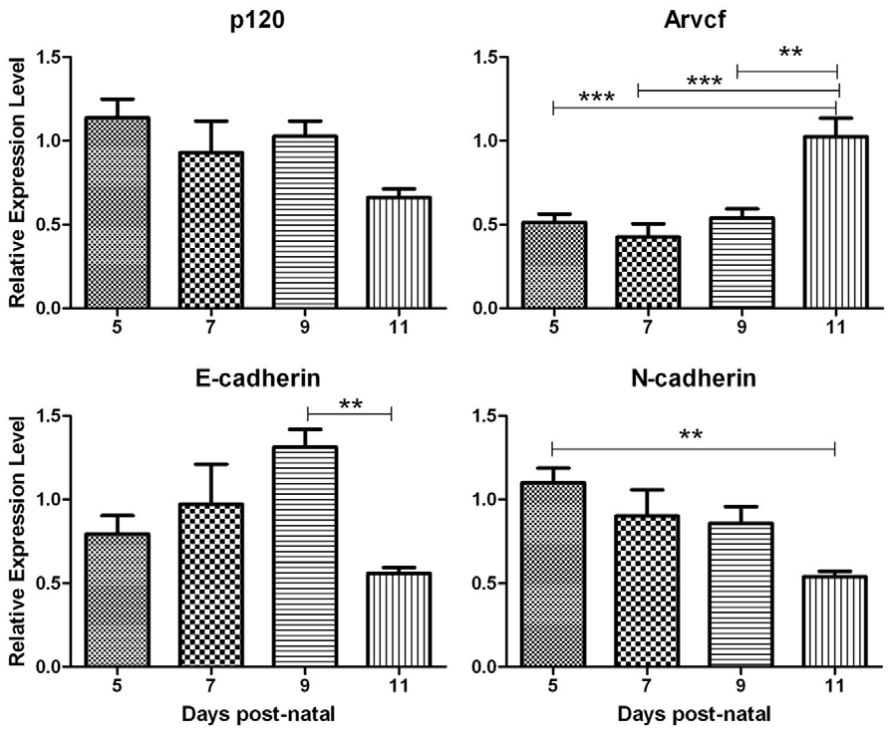
Secretory and maturation stage enamel organ express E- and N-cadherins, p120 catenin and *Arvcf*. qPCR was performed to identify the expression levels of adherens junction proteins in enamel organs responsible for dental enamel development. Expression was assessed in first molar enamel organs from mice at the indicated age. At days 5–7, enamel organs are predominantly at the secretory stage and at days 9–11, enamel organs are predominantly at the maturation stage of enamel development. Note that p120 is expressed at constant levels across these development stages. Arvcf is highly homologous to p120 and this is the first demonstration of its expression in the mammalian enamel organ. Each time point was performed in duplicate with RNA from six different enamel organs. *, P<0.05; **, P<0.01; ***, P<0.001.

To confirm that cadherins are expressed in tissues responsible for the developing mouse first molar, qPCR analysis was performed on first molar enamel organs (EO) at two specific stages of development. The results demonstrated that E-, and N-cadherins are each expressed during the secretory (P5-7) and maturation stage (P9-11) of enamel development (Fig. 1). Because p120 stabilizes cell surface cadherins, we also confirmed that p120-catenin was expressed in the developing enamel organ. p120 was expressed at a constant level from the secretory through the maturation stages of dental enamel development (Fig. 1). This suggests that cell surface cadherin stabilization is important during enamel formation. Because Arvcf is highly homologous to p120, we asked if Arvcf was also expressed in the mouse enamel organ. Expression of Arvcf was also observed in the first molar enamel organ during the secretory and maturation stages of development (Fig. 1).

### Conditional p120 Ablation Results in a Severe Enamel Phenotype

**Figure 2:**
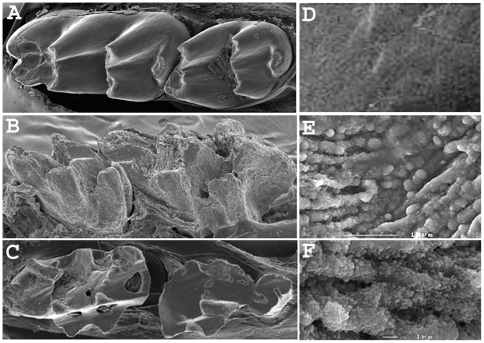
Enamel from K14-Cre p120-cKO mice is dysplastic. Scanning electron microscopy (SEM) of molars and enamel from wild-type (A, D) and K14-Cre p120-cKO mice (B, C, E, F). Note that the general shape of the cusps and fissures in the cKO mice is not altered. The dysplastic enamel on the cKO mouse molars (B) does not protect the teeth from abrasion as does normal highly mineralized enamel. The first molar from a six week old mouse (panel C, left) shows pulp chamber exposure due to excessive attrition. The tooth surface of the K14-Cre p120-cKO (E) consists of an unusual alignment of modular structures into distinct rows. Higher magnification shows the rows are composed of spherical structures of about 0.1 to 0.2 µm in diameter. The wild-type surface enamel is very smooth (D).

Conditional knockout (cKO) mice were generated by crossing mice containing floxed p120 with mice containing the keratin-14 promoter (K14) linked to *Cre* recombinase (K14-Cre p120-cKO) as previously described [21]. Keratin-14 is expressed in skin and in the enamel organ and the K14 promoter directs expression to these tissues [22]. The floxed region was purposefully extended so that *Cre* would not always recognize both floxed sites. This sometimes resulted in a mosaic distribution of normal cells with intact p120 and cells with ablated p120 in the same animal tissue. Scanning electron microscopy of molar teeth revealed severely malformed enamel in the K14-Cre p120-cKO mice (Fig. 2B, C, E, F). The surface layer of the cKO teeth (Fig. 2E, F) did not resemble normal enamel (Fig. 2D). High surface SEM magnification revealed spherical structures of about 1.0 µm in diameter. They were fused to form rows of what appear to be mineral deposits (Fig. 2E). The rows of fused spheres were composed of smaller spheres, about 0.1 µmin diameter (Fig. 2F). The spherical deposits on the tooth surface resembled amorphous calcium phosphate. Note that the general shape of the teeth was not affected (Fig. 2B). The cusps and fissures of the molar teeth were discernible. However, the surface material was not resistant to wear, as is normal enamel, and the teeth were rapidly abraded to reveal the pulp cavity (Fig. 2C).

**Figure 3:**
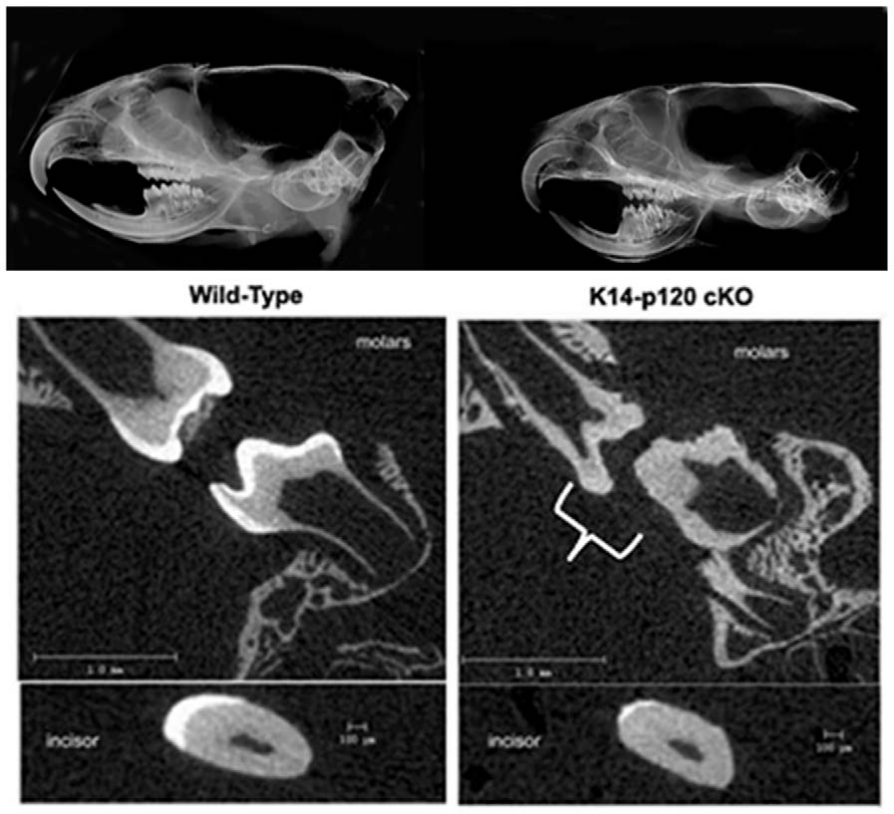
Enamel from K14-Cre p120-cKO mice does not mineralize properly. Faxitron analysis of wild-type and cKO mouse skulls reveals either absence of enamel or a thin layer of poorly mineralized enamel that is indistinguishable from the underlying dentin (Top). This assessment was corroborated by micro-CT analysis of molar and incisor teeth (Bottom). The wild-type molars had clearly defined opaque enamel layers whereas molars from the cKO mice did not (bracket identifies molars lacking a highly mineralized enamel layer). Note that small spots of more highly mineralized enamel are present on the cKO mouse molars. These data indicate that the globular material observed on the tooth surface by SEM is poorly mineralized. The cKO incisor tooth (bottom) had a thin layer of mineralized enamel covering a small portion of the labial tooth surface whereas the wild-type incisor had thick enamel covering the entire labial surface of the dentin.

Faxitron analysis of wild-type and K14-Cre p120-cKO mouse skulls revealed that the cKO mouse teeth had poorly mineralized enamel as observed by a decrease in x-ray opacity on the negative when compared to the wild-type teeth (Fig. 3, Top panel). This observation was confirmed by micro-CT analysis (Fig. 3, Bottom panels). In wild-type mice, a clear distinction was observed between the tooth enamel and dentin. The enamel covering the tooth surface was significantly more dense than the underlying dentin. In contrast, the cKO mice displayed very little difference in density between the enamel and dentin, indicating that the enamel had not properly mineralized. On close inspection, small white dots, indicative of spotty enamel mineralization, can be seen on the surface of the cKO molars (Fig. 3, Middle panel). These mineralized spots were consistent with the mineral spheres that were observed by SEM analysis of cKO surface enamel (Fig. 2E). These data demonstrated faulty enamel development in the K14-Cre p120-cKO mice, prompting further characterization.

### Enamel Organ Morphology is Dysplastic in K14-Cre p120-cKO Mice

**Figure 4:**
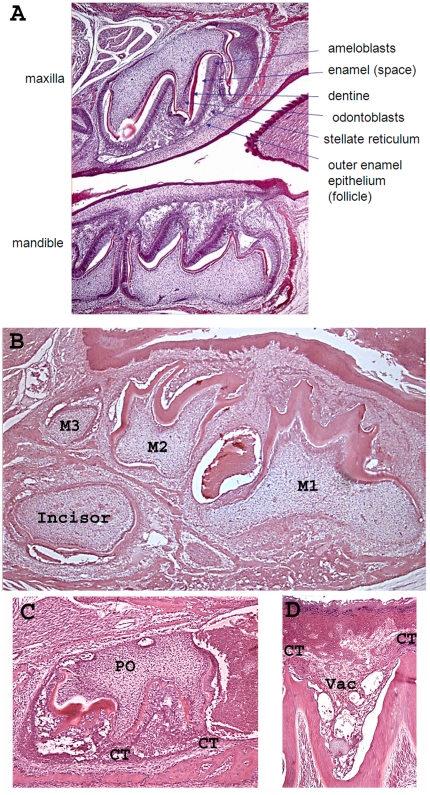
Molar enamel organs from K14-Cre p120-cKO mice are dysplastic and are adjacent to blood filled sinusoids. Wild-type mouse molars with identified tissue structures are presented (A) as a comparison to the molars from the cKO mice (B–D). Note that the enamel space (resulting from the demineralization of enamel) in the cKO mouse molars was greatly reduced in width (B) when compared to wild-type molars. Unusual large, blood-filled sinusoids were present, such as the one between the first (M1) and second (M2) molars in this section. The enamel organ morphology became dysplastic in the p120 ablated mice (C) and a high magnification (D) of an enamel organ between two cusp tips (CT) revealed the presence of clear vacuoles (Vac) where tissues were separated. With few exceptions, the ameloblasts, stratum intermedium, and stellate reticulum layers could not be definitively identified. A thick normal appearing layer of dentin was present in the cKO mouse molar teeth. PO, pulp organ.

Histological analysis of cKO molar enamel organs revealed that the enamel space was greatly reduced (Fig. 4B) when compared to wild-type controls (Fig. 4A). As shown in Figure 4B, a large blood-filled sinusoid was present between the first two molars (M2 and M1) in the cKO mouse section. These sinusoids were common around the developing molars of the cKO mice, but their exact location varied. Note that abnormal vacuoles were sometimes present where tissues of the enamel organ had separated from one another (Fig. 4C, D).

### The Ameloblast Layer of the K14-Cre p120-cKO Enamel Organ Become Disrupted near the Start of the Secretory Stage of Enamel Development

**Figure 5:**
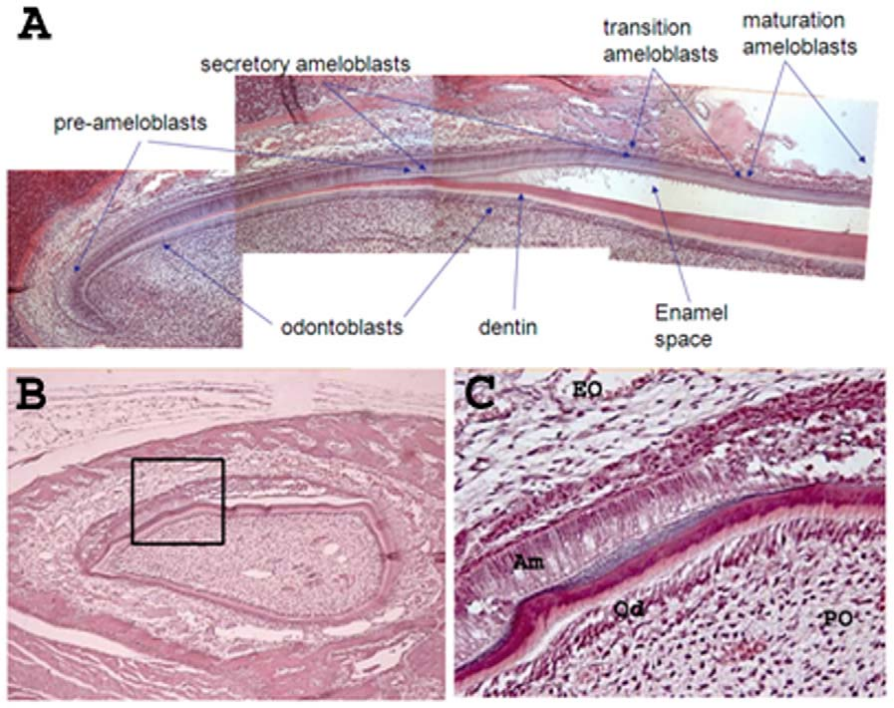
Incisor tooth development in K14-p120-cKO mice appears normal until the secretory stage of development when the ameloblasts flatten and become dysmorphic. A wild-type mouse incisor with identified tissue structures is presented (A) as a comparison for the incisor from the cKO mice (B, C). The p120 ablated incisor ameloblasts separate from the dentin surface prior to mineral formation (B). Panel C is a higher magnification of the boxed area in panel B. The p120 null ameloblasts (Am) abruptly alter their morphology soon after they enter the secretory stage of enamel development and become short flattened cells (C). The odontoblast (Od) and pulp organ (PO) appear normal in these teeth (C).

Morphological assessment of the wild-type continuously erupting mouse incisor (Fig. 5A) demonstrated that the initial stages of tooth development in cKO mice appeared unaffected (Fig. 5B, C). The cKO enamel organ had pre-ameloblasts that induced odontoblast differentiation. The odontoblasts secreted dentin matrix which then mineralized into dentin. However the ameloblasts changed shape beginning at the secretory stage where enamel matrix was expected. The ameloblasts became flattened and separated from the surrounding tissues (Fig. 5C). Therefore, the absence of p120 did not significantly disrupt incisor formation until the incisor reached the secretory stage of enamel development. This is when the ameloblasts move in rows and secrete enamel matrix to initiate enamel mineralization.

### Secretory Stage Ameloblasts Require p120-Catenin to Maintain Intercellular Adhesion

**Figure 6:**
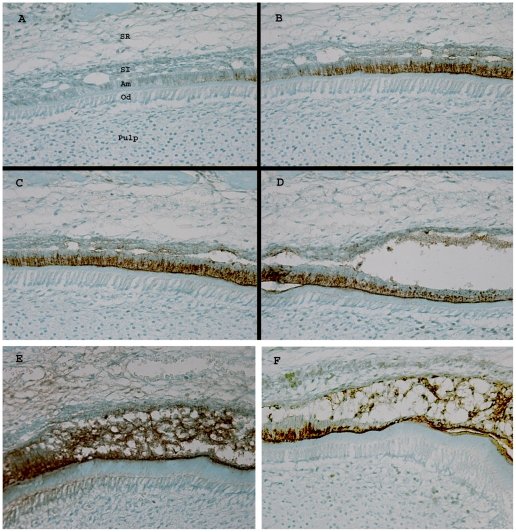
The p120 null ameloblasts detach from the incisor dentin surface, the stratum intermedium, and from each other. Secretory ameloblasts (Am) are identified by positive immunostaining for amelogenin. Panels A–D represent sequential sections of an incisor from a cKO mouse. Pre-secretory ameloblasts do not express amelogenin (A) whereas secretory stage ameloblasts do express amelogenin (B–D). Note the vacuoles above the ameloblasts in the stratum intermedium (SI) that grow progressively larger as development progresses. The right side of panel D shows ameloblasts that have completely lost contact with the stratum intermedium. However, the ameloblasts also lose contact with the dentin surface and with each other (E, F). The ameloblasts in panels E and F are disorganized and appear to have lost most but not all cell adhesion properties so they appear stretched between the stratum intermedium and dentin. Od, odontoblast; SR, stellate reticulum.

Only secretory stage ameloblasts express appreciable levels of the enamel matrix protein amelogenin. Therefore, incisors from cKO mice were extracted, paraffin embedded and processed for amelogenin immunostaining (Fig. 6). This served to definitively label ameloblasts so that their developmental progression could be more easily observed during the secretory stage of enamel development. Figure 6, panels A–D show the sequential progression of ameloblast development, starting with pre-secretory stage ameloblasts that do not express amelogenin (Fig. 6A), and ending with secretory stage ameloblasts that have detached from the adjacent stratum intermedium (Fig. 6D). Note that vacuoles (areas of detachment) appeared between the stratum intermedium and ameloblasts during the presecretory stage (Fig. 6A) and that these vacuoles became progressively larger as development proceeded (Fig. 6B–D). The vacuoles did not contain amelogenin and the adjacent ameloblasts had basal nuclei. Therefore the ameoblasts were still polarized during the early secretory stage. Depolarization occurred later when the ameloblasts also detached from the dentin surface and from each other (Fig. 6E, F). The ameloblasts in these last two panels appear to have lost most but not all intercellular adhesion. They appear loosely connected to from a randomly oriented meshwork that stretches between the dentin surface and the stratum intermedium. In contrast, wild-type secretory stage-ameloblasts maintain a highly oriented, tall, columnar morphology (Fig. 5A). We therefore asked if p120 ablation affected ameloblast cell surface cadherin localization.

### The Presence of Appreciable N-Cadherin in Ameloblasts Requires p120 Expression

**Figure 7:**
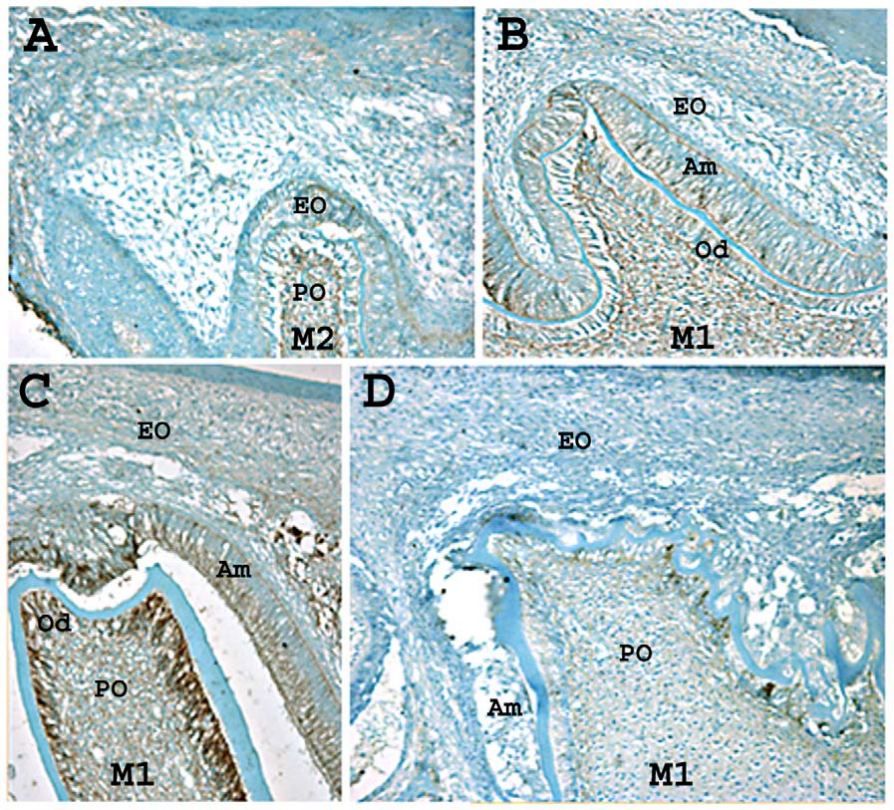
N-cadherin is expressed in wild-type secretory stage ameloblasts, but not in p120 ablated ameloblasts. In the less mature second molar (M2), N-cadherin was not expressed (A) in the enamel organ (EO) or pulp organ (PO) of three day-old mice. In the more mature first molar (M1), N-cadherin was expressed (B, C). After dentin matrix deposition, odontoblasts (Od) and ameloblasts (Am) showed lateral membrane immunostaining for N-cadherin, and the apical and basal terminal web apparatus of ameloblasts were also stained positively (B). After enamel matrix deposition, the odontoblasts stain intensely (C). A developing cusp tip from the first molar of a P3 K14-Cre p120-cKO mouse stained for N-cadherin (D). N-cadherin expression was detected in odontoblasts, but not in the ameloblasts from this molar. Note that the dentin appears rough and mildly dysplastic.

The mouse molars shown in Figure 7 are postnatal day 3 (P3). At this age ameloblasts from second molars are at the pre-secretory stage and ameloblasts from first molars are predominantly at the secretory stage of enamel development (Fig. 7A, B). Immunohistochemical analysis of molar teeth demonstrated that N-cadherin was expressed in ameloblasts and odontoblasts but not in other odontogenic cells (Fig. 7C). N-cadherin expression was initiated after pre-odontoblasts and pre-ameloblasts had fully differentiated and were producing their respective matrices (Fig. 7B, C). However in cKO mice, N-cadherin was detected in odontoblasts, but not in ameloblasts present in the first molar cusp tip (Fig. 7D). This result suggests that p120 plays a role in cadherin cell surface stabilization within ameloblasts of the developing enamel organ.

### Incomplete p120 flox Region Removal Causes a Mosaic Pattern of Adjacent Normal and Malformed Ameloblasts and Associated Enamel

**Figure 8:**
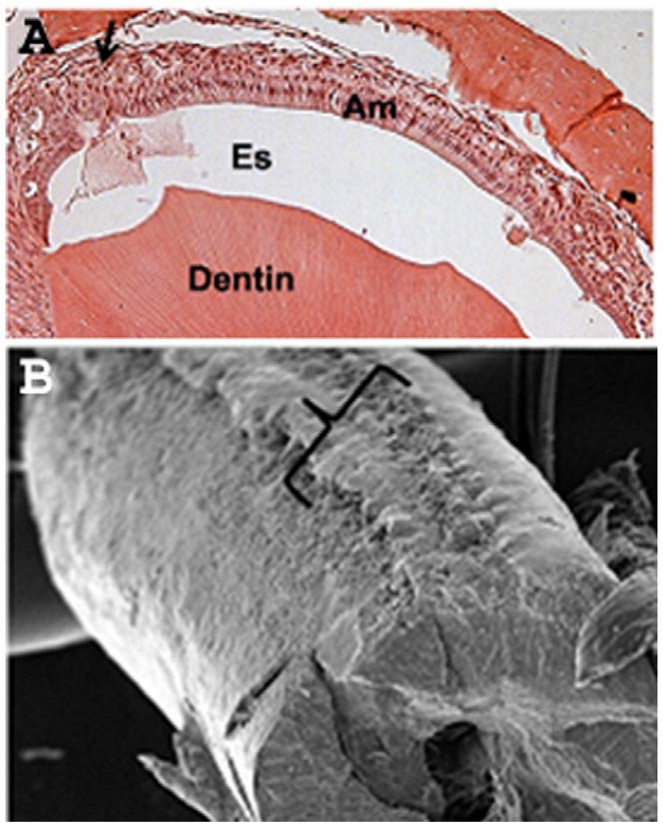
Mosaic phenotype of the K14-Cre p120-cKO mouse incisor. A section through a mosaic cKO enamel organ (A) showing normal ameloblasts (Am) in the middle and malformed ameloblasts to the left (arrow). Es, enamel space. SEM analysis of the same cKO mosaic incisor (B). The right side of the bracket touches normal enamel and the left side touches an abnormal, enamel-free area. Between the two sides of the bracket is the malformed dysplastic enamel. Note that the cKO mouse is capable of forming normal enamel. Therefore, the observed enamel dysplasia is not a secondary effect of conditional p120 ablation.

**Figure 9:**
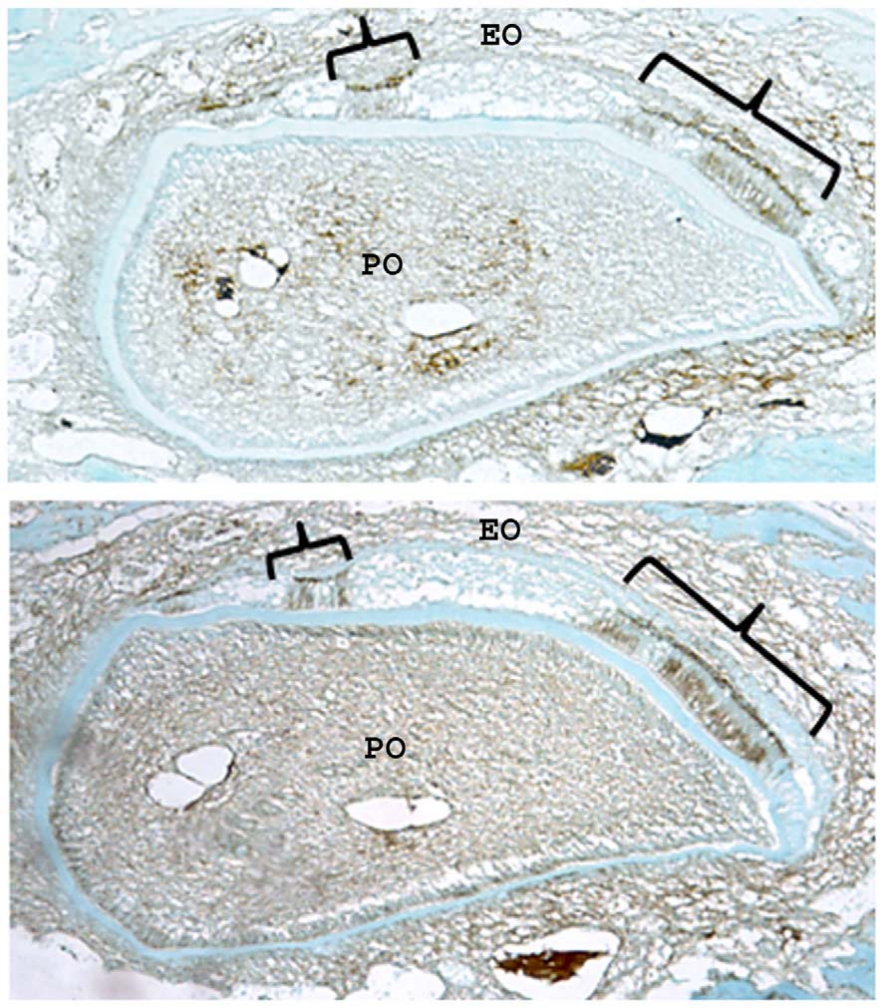
E-cadherin is only expressed in ameloblasts capable of expressing p120. In the same K14-Cre p120-cKO mosaic incisor shown in Figure 8, E-cadherin (top) and p120 catenin (bottom) was immunolocalized in adjacent cross-sections. E-cadherin is expressed exclusively in normal appearing ameloblasts (brackets) that also express p120. However, in flattened, malformed ameloblasts where p120 was ablated, immunostaining for E-cadherin was not detectable. EO, enamel organ; PO, pulp organ.

Mosaic mice were generated as a control to evaluate the effect of presence and absence of p120 within the same tissue. As mentioned above, this was accomplished by placing an extended floxed region within the p120 gene so that the *Cre* recombinase would not always remove the floxed DNA, resulting in sporadic normal p120 expression. Morphological analysis of a mouse incisor enamel organ revealed a malformed array of stacked ameloblasts beside normal columnar ameloblasts located in a single row (Fig. 8, Top panel). Analysis of a single mosaic mouse incisor by SEM revealed a normal smooth appearing enamel surface located next to rough dysplastic enamel which in turn was located adjacent to an area of missing enamel (Fig. 8, Bottom panel). These data (Figs. 8, 9) demonstrate that localized deletion of p120 caused cadherin loss, dysplastic ameloblasts, and malformed enamel in mice that were otherwise capable of proper dental enamel formation.

To show that p120 is responsible for maintaining the presence of E-cadherin in ameloblasts, E-cadherin expression was examined in a mouse incisor that was mosaic for p120. Immunohistochemical staining of adjacent incisor cross sections for E-cadherin or p120 demonstrated colocalization of these proteins in tall, columnar ameloblasts that appear normal (Fig. 9). However, in flattened, malformed ameloblasts where p120 was ablated, immunostaining for E-cadherin was undetectable. This demonstrates, within the same mosaic incisor, that the presence of E-cadherin in ameloblasts is dependent on p120 expression. These data are supportive of p120 playing an important regulatory role in intercellular adhesion during the secretory stage of enamel development when rows of ameloblasts slide by one another.

## Discussion

We have used conditional p120 ablation in mouse epithelial tissues to examine the loss of p120 on tooth and dental enamel development. The results reveal that p120 is essential for normal dental enamel formation. Ablation of p120 results in striking defects in cell-cell adhesion and ameloblast morphology. Remarkably, p120 ablation did not significantly affect the ameloblasts responsible for dental enamel formation until the ameloblasts reached the secretory stage of development. This was surprising because during early tooth development reciprocal epithelial and mesenchymal signaling is required for teeth to continue development as the overall shape of the tooth is defined [24], [25], [26]. Although the dentin from the cKO mice was mildly dysplastic (Fig. 7D), the overall shape of the teeth was normal (Figs. 2, 3) indicating that p120 is dispensable in epithelium during early tooth development.

Another striking feature of the cKO mouse teeth was that the pre-ameloblasts began separating from the stratum intermedium and this separation grew more prominent as development progressed to the secretory stage (Fig. 6 A–D). In specific instances during the secretory stage, the ameloblasts separated from both the dentin surface and the stratum intermedium, but remained loosely attached to each other. This resulted in a random meshwork pattern of cells that spanned between the enamel and stratum intermedium (Fig. 6E, F). It is during the secretory stage that ameloblasts start moving in rows relative to one another to begin forming the decussating rod pattern that is characteristic of rodent enamel [3], [4], [5]. Therefore, cell attachments must be fine-tuned to maintain the moving ameloblast row while simultaneously allowing an adjacent row to detach and move in a different direction. It is notable that adherens junctions were identified along the possible sliding interface of adjacent ameloblast rows [18]. We speculate that p120 plays an important role in directing this elegant process and that in the absence of p120, ameloblast cell-cell attachment and detachment becomes disorganized resulting in the loss of ameloblast organization as a tall columnar cell layer. This concept is consistent with the observed thin layer of poorly formed nodular-looking dental enamel that forms on the p120 ablated mouse molars (Fig. 2). The disorganized ameloblasts would be expected to form a disorganized enamel layer. Because p120 was previously demonstrated to stabilize cell surface cadherins [13], [14], [16], [20], [27] and lack of p120 may be responsible for ameloblast detachment, we investigated the possibility that p120 ablated ameloblasts have reduced cadherin protein levels.

Three cadherin genes (*CDH*) were previously demonstrated as expressed in the developing enamel organ [17], [28], [29], [30], [31], [32]. These three cadherin genes encode E-cadherin (*Cdh1*), N-cadherin (*Cdh2*), and P-cadherin (*Cdh3*). Here we demonstrate that both E- and N-cadherins are reduced in ameloblasts with p120 ablation (Figs. 7, 8). These data are consistent with a role for p120 in stabilizing the cell surface localization of these cadherins as was demonstrated previously for E- and P-cadherins in mouse salivary gland [19] and epidermis [21]. Arvcf had not previously been identified in the enamel organ (Fig. 1). Its expression was of interest because it is highly homologous to p120 and *in vitro* it can rescue cadherin stability in p120 deficient cell lines [13]. However, although Arvcf may have supported early tooth development in the cKO mice, it did not adequately substitute for p120 function in secretory-stage ameloblasts. Our results suggest that cadherins are important for secretory stage ameloblast cell layer organization and enamel development.

An additional unexpected feature of the p120 ablated mouse was the abnormal presence of sinusoids located at various positions adjacent to the developing teeth (Fig. 4B). The sinusoids are not merely separated tissues. They have a defined endothelial cell lining and contain red blood cells. How these sinusoids arise within the enamel organ is unknown. However, in addition to stabilizing cadherins, p120 can also regulate cell signaling by interacting with the POZ family transcription factor, Kaiso [33], [34], [35] and by influencing the activity of Rho GTPases [36], [37], [38]. In addition, p120/Kaiso was demonstrated to play a functional role in canonical and noncanonical Wnt signaling [34], [39]. Perhaps, the absence of p120 alters cell signaling in such a way as to promote the formation of sinusoids in the enamel organ. This would implicate p120 in a previously unknown function.

Five other studies have assessed conditional knockout of p120 in mouse tissues. These studies demonstrated that p120 mediated inflammatory responses in the skin [21], that salivary gland development was dysmorphic and that acinar cell development was blocked [19], that spine and synapse densities along dendrites were dramatically reduced [20], that p120 regulates keratinocyte mitosis and its absence promotes tumor formation [27], and that p120 enhances endothelial proliferation and periocyte coverage of developing microvessels [40]. In concordance with our results, each study showed markedly reduced levels of cadherins in their respective tissues. Although a marked reduction in adherens junction components were observed in the skin, this did not appear to grossly alter the desmosomes, tight junctions, or the sealing of intercellular membranes within the epidermis [21]. This is consistent with our results since tooth development progressed with normal tissue differentiation up until the secretory stage of enamel development and supportive intercellular interactions are required for this to occur [41].

In wild-type mice, the normal submandibular gland consists mostly of secretory acini, which are responsible for making saliva. Acini are organized into lobules and are drained by ducts into the oral cavity. The p120 ablated salivary glands had severe defects in cell adhesion, cell polarity, and epithelial morphology. They lacked acini and consisted entirely of misshapen occluded ducts [19]. The secretory stage ameloblasts and submandibular acini are both high volume protein secretory cells. However, in contrast to the tooth, the abnormalities observed in the submandibular gland coincided with p120 ablation and the acini never formed whereas the ameloblasts did attain their tall columnar shape prior to detachment from surrounding tissues.

Finally, it was significant that normal enamel could form in the mosaic incisors (Fig. 9). Enamel formation is sensitive to environmental influences. For example, persistent high fever will result in enamel defects in the rat incisor [42]. However, because the mosaic mouse was capable of forming normal enamel, we can conclude that secondary effects were not the cause of the enamel malformation observed in the K14-Cre p120-cKO mice.

### Summary and Significance

Taken together, these data demonstrate that: p120 is dispensable in epithelium during early tooth development, p120 stabilizes ameloblast cadherins, p120 may direct ameloblast cell movement in rows, the absence of p120 may alter cell signaling in such a way as to promote the formation of sinusoids, and that *in vivo*, p120 plays a critical role in maintaining the tall, columnar organization of secretory stage ameloblasts.

## Materials and Methods

### Ethics Statement

All animals used in this study were housed in an Association for Assessment and Accreditation of Laboratory Animal Care (AAALAC) accredited facilities (animal welfare assurance number: A3051-1) and were treated humanely, based on a protocol (08-019) approved by the Institutional Animal Care and Use Committee (IACUC) at The Forsyth Institute. Experimental protocols were designed with institutional and National Institutes of Health guidelines for the humane use of animals. The K14-Cre p120-cKO mice were generated as described previously [21]. C57BL/6 mouse first molar enamel organs were extracted for quantitative real-time PCR analysis.

### Quantitative Real-time PCR (qPCR)

mRNA was extracted from 5-11 day-old first molar enamel organs and reverse transcribed for qPCR analysis. PCR temperature profile was 3 min 95°C initial melt then; 20 s 95°C, 30 s 62–64°C for 45 cycles then 30 s 95°C, for 1 cycle; 1 min 55°C followed by stepwise temperature increases from 55°C to 95°C to generate the melt curve. Standard curves were generated with each primer set using control cDNA preparations and a 10-fold dilution series ranging from 1000 ng/ml to 100 pg/ml. PCR efficiencies and relative expression levels of experimental gene expression as a function of *Ef1α1*, *Actb* (β-actin), and *Gapdh* control gene expression were calculated as previously described [43]. *Arvcf* (PrimerBank ID 15628021a1) and *p120-catenin* (PrimerBank ID 6671686a3) primers (Table 1) were designed by Primerbank [44], [45]. *E-cadherin* and *N-cadherin* were designed as listed in the Roche Universal Probe Library Assay Design Center (http://qpcr.probefinder.com/organism.jsp). Each time point represents duplicate qPCR analysis of mRNA extracted from six different enamel organs. Statistical significance was assessed by one-way ANOVA followed by Tukey post-test.

**Table 1:** Primers used for quantitative real-time PCR (qPCR).

	GenBank ID #	5′ Primer	3′ Primer	°C
p120	NM_007615.4	GGGTCTCACCACAAG ATGCC	TCCTGGGGTCCGTTGAGTTT	63
Arvcf	NM_033474.2	GGCTGGGAGCTAGAGCCTAA	GTTCCGCATGATGCACACAC	62
Cdh1(E-cad)	NM_009864	CAGCCTTCTTTTCGGAAGACT	GGTAGACAGCTCCCTATGACTG	62
Cdh2 (N-cad)	NM_007664.4	CCAGCAGATTTCAAGGTGGAC	TTACAGCTACCTGCCACTTTTC	62
Ef1α1	NM_010106	ATTCCGGCAAGTCCACCACAA	CATCTCAGCAGCCTCCTTCTCAAAC	62
Actb	NM_007393	TGACGGCCAGGTCATCACTATT	ACCCAAGAAGGAAGGCTGGAAA	64
Gapdh	NM_008084	GCAAAGTGGAGATTGTTGCCAT	CCTTGACTGTGCCGTTGAATTT	62

### X-ray Analysis and Scanning Electron microscopy (SEM)

For X-ray analysis, adult wild-type and K14-Cre p120-cKO mouse heads were placed in a Hewlett Packard Faxitron 43855a X-Ray system for 20 min at 40 kV using Kodak so-253 high speed holographic film. For SEM, erupted molar and incisor teeth were air-dried, fastened to stubs, sputter coated, and examined using a JEOL 6400 scanning electron microscope.

### MicroComputed Tomography (micro-CT)

First molar and incisor dental enamel was assessed for level of mineralization by micro-CT performed on adult wild-type and K14-Cre p120-cKO mice. Skulls were immersed in saline and scanned (μCT-40; Scanco Medical) with the following settings 70 kV, 114 µA, and 0.012 mm isotropic voxels. After reconstruction, normal, highly mineralized enamel appeared more translucent than the dentin and surrounding bone.

### Histology

Heads were obtained from euthanized wild type and K14-Cre p120-cKO mice at ages 3, 7, 9, and 15 days. The tissues were fixed in 10% zinc formalin overnight, washed with PBS and, in order to facilitate the sectioning of highly mineralized tissues, the jaws were decalcified in a 1∶1 solution of 20% sodium citrate and 10% formic acid for 2 weeks. This and all subsequent incubations were performed at ambient temperature. The tissues were dehydrated in a graded series of ethanol washes and embedded in paraffin for sectioning. Deparaffinized and rehydrated sections were stained with haemotoxylin/eosin.

### Immunohistochemistry

Dewaxed and rehydrated sections from mouse jaws were subjected to heat activated antigen retrieval (55°C) in 10 mM sodium citrate, pH 6. Endogenous peroxidase was quenched in 3% hydrogen peroxide in methanol. Sections were incubated in blocking agent for 20 min followed by overnight incubation in 1∶10,000 diluted antisera specific for amelogenin (Abcam, Cambridge, MA), N-cadherin (Invitrogen, Carlsbad, California), or in 1∶250 diluted p120-catenin antisera (BD Biosciences, San Jose, CA). Staining was visualized by incubation in Vectastain Elite ABC (Vector Laboratories, Burlingame, CA) peroxidase-conjugated antibody and Sigma Fast 3,3′-diaminobenzidine substrate (Sigma,St. Louis, MO). Sections were counterstained with 0.1% Fast Green and examined by light microscopy. Negative control sections excluded the primary antisera but were otherwise treated the same as experimental sections.
